# Genetic characterisation and phylogenetic status of whipworms (*Trichuris* spp.) from captive non-human primates in China, determined by nuclear and mitochondrial sequencing

**DOI:** 10.1186/s13071-018-3100-5

**Published:** 2018-09-20

**Authors:** Yue Xie, Bo Zhao, Eric P. Hoberg, Mei Li, Xuan Zhou, Xiaobin Gu, Weimin Lai, Xuerong Peng, Guangyou Yang

**Affiliations:** 1 Department of Parasitology, College of Veterinary Medicine, Sichuan Agricultural University, Chengdu, 611130 China; 2 Chengdu Zoo, Chengdu, 610081 Sichuan China; 3 Division of Parasitology, Museum of Southwestern Biology, University of New Mexico, Albuquerque, NM 87131 USA; 4 Institute of Animal Genetics and Breeding, College of Animal Science and Technology, Sichuan Agricultural University, Chengdu, 611130 China; 5 Department of Chemistry, College of Life and Basic Science, Sichuan Agricultural University, Chengdu, 611130 China

**Keywords:** Primates, Whipworms, *Trichuris*, Genetic analysis, Phylogeny, Nuclear ITS, Mitochondrial *cox*1

## Abstract

**Background:**

Whipworms (Nematoda: Trichuridae), among the most common soil-transmitted helminths (STHs), can cause the socioeconomically important disease trichuriasis in various mammalian hosts including humans and non-human primates. For many years, *Trichuris* from non-human primates has been assigned to the same species as the one infecting humans *Trichuris trichiura*. More recently, several molecular reports challenged this assumption following recognition of a *Trichuris* species complex observed in humans and non-human primates. A refined concept for species limits within *Trichuris* contributes to an understanding of diversity and the potential (zoonotic) transmission among humans and non-human primates. In this study, we expanded previous investigations by exploring the diversity of *Trichuris* among eight primates including three Asian autochthonous species (i.e. *Rhinopithecus roxellana*, *Rhinopithecus bieti* and *Nomascus leucogenys*). Species-level identification, whether novel or assignable to known lineages of *Trichuris*, was based on analyses of nuclear internal transcribed spacers (ITS) and mitochondrial cytochrome *c* oxidase subunit 1 (*cox*1) genes.

**Results:**

In total, seven genetically distinct subgroups of whipworms were determined to be present among the primates sampled. Most *Trichuris* lineages, including Subgroups 1, 1’, 3, 5 and 6, showed a broad host range and were not restricted to particular primate species; in addition to *T. trichiura*, a complex of *Trichuris* species was shown infecting primates. Furthermore, it was assumed that *Trichuris* spp. from either *N. leucogenys* and *P. hamadryas* or *R. roxellana* and *R. bieti*, respectively, were conspecific. Each pair was indicated to be a discrete lineage of *Trichuris*, designated, respectively, as Subgroups 1 or 1’ and 2, based on integrated genetic and phylogenetic evidence.

**Conclusion:**

These results emphasise that the taxonomy and genetic variations of *Trichuris* are more complicated than previously acknowledged. These cumulative molecular and phylogenetic data provide a better understanding of the taxonomy, genetics and evolutionary biology of the whipworms.

**Electronic supplementary material:**

The online version of this article (10.1186/s13071-018-3100-5) contains supplementary material, which is available to authorized users.

## Background

Whipworms (Nematoda: Trichuridae) are among the most common soil-transmitted helminths (STHs) and cause trichuriasis in various mammalian hosts with important socioeconomic impact [1]. Humans and non-human primates, as well as mammals such as ruminants, marsupials and rodents, serve as definitive hosts [2–5]. Infection with *Trichuris* is based on direct transmission and generally results from oral ingestion of the infective eggs found in food, water and soil [6]. Following ingestion, the first-stage larvae (L1s) hatch and travel to the large intestine (caecum and colon) of mammalian hosts where they burrow into the intestinal mucosa and further develop, moult and propagate as dioecious adults that release eggs into the faeces. Damage to the intestinal mucosa is caused by the larval and adult worms that can lead to a variety of symptoms including typhlitis, colitis, bloody diarrhoea, growth stunting and increased susceptibility to other pathogens [7].

Currently, about 80 valid species are identified in the genus *Trichuris*, with most considered specific to particular taxonomic groups of hosts [8]. Among them, both species of *Trichuris*, namely *Trichuris trichiura* (Linnaeus, 1771) and *Trichuris suis* Schrank, 1788, are regarded as zoonotic parasites and pose threats to public health [9]. Traditional taxonomic sampling and research have focused on characterisation and differentiation of human-originated *T. trichiura* and pig-originated *T. suis* with the conclusion that these nominal taxa of whipworms are two separate but closely related species [10–13]. Non-human primates, the closest human relatives, can endure high whipworm infections that are typically assumed to be *T. trichiura* according to morphological criteria [14–18]. This assumption appears reasonable because *T. trichiura* has previously been found in such primates as macaques (*Macaca mulatta* Zimmerman, 1780), crab-eating macaques (*Macaca fascicularis* Raffles, 1821) and other Old and New World monkeys [19–22].

Several recent molecular studies, however, revealed that primates including humans might host more than one species of *Trichuris*. For instance, Ravasi and colleagues [23] found two distinct *Trichuris* species occurring, respectively, in humans and baboons (*Papio ursinus* Kerr, 1792) based on sequencing the nuclear internal transcribed spacers (ITS, ITS1-5.8S-ITS2). Further, Callejón et al. [24] confirmed *Trichuris* sp. of *P. ursinus* as a new species, *Trichuris ursinus* Callejón, Halajian & Cutillas, 2017, using biometrical and molecular evidence. Likewise, Hansen et al. [25] showed that humans and baboons (*Papio anubis* Lesson, 1827 and *Papio hamadryas* Linnaeus, 1758) were hosts for *Trichuris* species representing distinct lineages based on differentiation involving sequence analysis of a combination of ITS2 and β-tubulin genes. Furthermore, Ghai et al. [26] suggested that the whipworms infecting diurnal monkey species, chimpanzees and humans may comprise three genetically distinct *Trichuris* groups, with one group showing broad host range and others with a narrow spectrum of recognised hosts. Additionally, building on datasets using complete mitochondrial DNA (mtDNA), Liu et al. [27] identified a potentially novel *Trichuris* sp. in a non-human primate François’ leaf-monkey. Another study by Hawash et al. [28], employing comparative mitogenomics, showed that multiple species of *Trichuris* inhabit humans and baboons. Considering that cross-infection and possible hybridisation have been documented between *Trichuris* spp. [12, 29], there remains considerable controversy concerning whether putative lineages in primates constitute one or more species [3]. Resolution of species limits can contribute to a stable taxonomy for species of *Trichuris* and therefore provides a pathway to identify the nature of host range among non-human primates and humans and the potential for zoonotic transmission.

Ongoing epidemiological surveys on potential helminthic zoonoses, centred in the southwestern zoos of China, expanded previous investigations by including specimens of *Trichuris* in eight species of primates including three autochthonous to Asia (i.e. *Rhinopithecus roxellana* Milne-Edwards, 1870; *Rhinopithecus bieti* Milne-Edwards, 1897; and *Nomascus leucogenys* Ogilby, 1840). Our goal was to characterise whipworms from an assemblage of diverse hosts relative to currently defined lineages of *Trichuris* facilitating (i) identification of these isolates at species level by genetically analyzing the nuclear ITS and mitochondrial cytochrome *c* oxidase subunit 1 (*cox*1) markers; and (ii) determination of levels of genetic variation among these *Trichuris* by comparisons among those documented in other non-human primates and humans which are available in GenBank.

## Methods

### Animals and parasite sampling

**Fig. 1:**
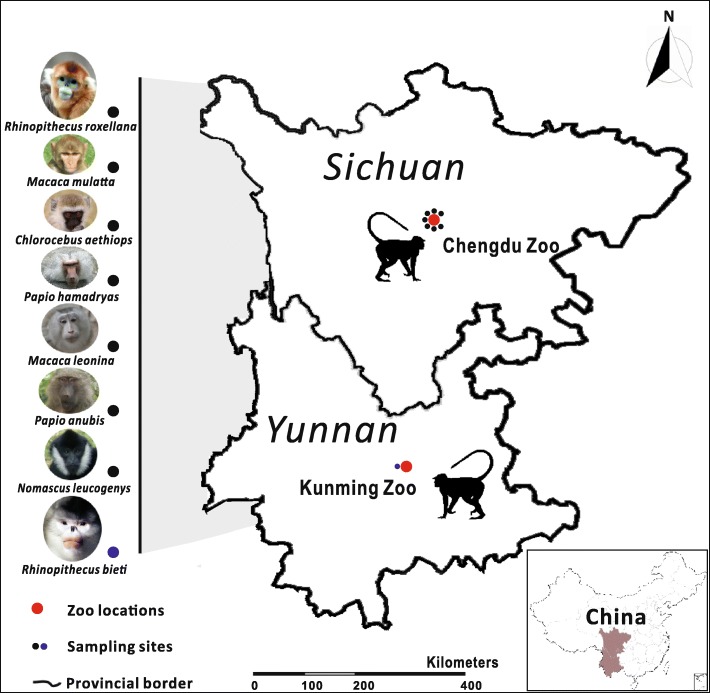
Provincial map of China showing the sampling location (small black circles) of *Trichuris* spp. collected from eight distinct non-human primates in Chengdu Zoo in Sichuan Province and another in Kunming Zoo in Yunnan Province (large red circles)

A total of eight non-human primate host species, including the golden snub-nosed monkey (*R. roxellana*), black snub-nosed monkey (*R. bieti*), vervet monkey (*Chlorocebus aethiops* Linnaeus, 1758), rhesus monkey (*M. mulatta*), northern white-cheeked gibbon (*N. leucogenys*), northern pig-tailed macaque (*Macaca leonina* Blyth, 1863), anubis baboon (*P. anubis*) and hamadryas baboon (*P. hamadryas*) from the Chengdu Zoo (Sichuan, China) and Kunming Zoo (Yunnan, China) were sampled in this study (Fig. 1). These animals all were bred in the zoo conditions and socially raised and caged in groups, in strict accordance with good animal practices and veterinary inspection procedures. No introductions and/or translocations were permitted during the captive period. After treatment with mebendazole 20 mg/kg daily for 3 days (B. Zhao unpublished data), the animals were temporarily separated and then 18 adult whipworm specimens were collected from naturally infected golden snub-nosed monkeys, northern white-cheeked gibbons, anubis baboons, vervet monkeys, northern pig-tailed macaques, rhesus monkeys and hamadryas baboons (Table 1). Adult worms from each host were washed separately in phosphate-buffered saline, morphologically identified to the genus level [30, 31], fixed in 70% (v/v) ethanol and then stored at -20 °C until use. Whipworm eggs from faeces of two black snub-nosed monkeys were also isolated for this study because no worms were found during and after anthelmintic treatment. All non-human primate groups were sampled only once to prevent pseudo-replication of individuals. All samples were collected between January and November 2016.

**Table 1:** Summary information of non-human primate hosts and their *Trichuris* parasites sampled in this study

Host species	Sampled numbers	
	Host	Parasite^a, b^
Golden snub-nosed monkey (*Rhinopithecus roxellana*)	1	1 (1)
Anubis baboon (*Papio anubis*)	3	4 (1/1/2)
Vervet monkey (*Chlorocebus aethiops*)	1	2 (2)
Northern pig-tailed macaque (*Macaca leonina*)	1	3 (3)
Rhesus monkey (*Macaca mulatta*)	2	4 (2/2)
Northern white-cheeked gibbon (*Nomascus leucogenys*)	1	2 (2)
Hamadryas baboon (*Papio hamadryas*)	2	2 (1/1)
Black snub-nosed monkey (*Rhinopithecus bieti*)	2	2 (1/1)

^a^Parasite numbers sampled from each individual are shown in parentheses

^b^*Trichuris* eggs from faeces of two black snub-nosed monkeys were included in this study

### Sample processing and DNA isolation

Morphology separated male and female worms. The whole male worms were used to extract genomic DNA whereas only the thin anterior part (3/5) of female worms was used for DNA isolation, as described elsewhere [12, 25, 28]. For faecal eggs, a modified ethyl acetate sedimentation method was used for egg purification and concentration [32], followed by egg-shell breakage with an alternate boiling and freezing (100 °C and liquid nitrogen) procedure for several cycles. Worm DNA was isolated using the Universal Genomic DNA Extraction Kit Ver. 3.0 (TaKaRa Biotech, Dalian, China), and egg DNA was isolated from 200 μl of sedimented faeces using the QIAamp DNA Mini Stool Kit (Qiagen, Hilden, Germany), followed by further purification with the MasterPure DNA Purification Kit (Epicentre, Madison, USA) according to the manufacturer’s instructions. DNA purity and concentration were detected using a NanoDrop ND-2000 (Thermo Scientific, Wilmington, DE, USA).

### PCR amplification and sequencing

The nematode parasite nuclear ITS and mitochondrial *cox*1 genes were amplified to verify these *Trichuris* isolates using polymerase chain reaction (PCR). On the basis of the alignments of the relatively conserved regions of the congeneric species *T. trichiura*, *T. suis* and *Trichuris muris* in GenBank^TM^ (see Table 2), the full ITS region (~1200 bp) and a fragment (~800 bp) of the *cox*1 gene were separately amplified from individual DNA samples with newly designed primers. Two PCR primer sets were as follows: ITS, forward (5'-ATC AGA ACA CAG CAA CAG-3') and reverse (5'-AAC ATC GAG GAG ACG TAC-3'); *cox*1, forward (5'-AAA AAT GGC TAT ATA CAG GT-3') and reverse (5'-GGG GCC AAA TAC TTT AAA T-3'). All PCR reactions were conducted in a total volume of 50 μl containing 15–20 ng of genomic DNA (gDNA), 25 μl 2× Phusion High-Fidelity PCR Master Mix (Finnzymes, Espoo, Finland), 3 μl gDNA, 3 μl of each primer (10 pmol/μl; TaKaRa) and 16 μl of ddH_2_O. Thermal cycling conditions carried out in a Mastercycler Gradient 5331 thermocycler (Eppendorf, Hamburg, Germany) were an initial denaturation at 94 °C for 5 min; then for ITS, 35 cycles of 94 °C for 30 s, 45.2 °C for 30 s and 72 °C for 45 s; but for *cox*1, 35 cycles at 94 °C for 30 s, 49 °C for 30 s and 72 °C for 45 s; followed by a final step at 72 °C for 10 min. For each amplification, samples without parasite gDNA and host DNA as negative controls were also included to test possible contamination. All PCR products were examined on agarose (1%) gels to verify that they represented the target bands. The corrected gel-isolated amplicons were column-purified with the TIANgel Midi Purification Kit (TIANGEN, Beijing, China) and sub-cloned into *Escherichia coli* (DH5a) using the vector pGEMH-T (Promega, Madison, WI, USA). Positive clones were screened and sent for sequencing (Invitrogen Biotechnology Co. Ltd., Shanghai, China) using the T7 and SP6 primers. To ensure maximum accuracy, an individual clone from each specimen was sequenced three times independently. The consensus sequences were used for the following bioinformatic analyses and deposited in GenBank under the accession numbers KT344825-KT344832 and MH390359-MH390370 for ITS and KT344817-KT344824 and MH390703-MH390714 for *cox*1.

**Table 2:** Summary information of *Trichuris* and outgroup species used for molecular identification in the present study

*Trichuris* species	Stages	Host species	Country		Living conditions	GenBank ID		Reference
			ITS	*cox*1		ITS	*cox*1	
*Trichuris colobae*	Eggs	Colobus monkey	Spain	–	Zoo	FM991956	–	Cutillas et al. [11]
*Trichuris colobae*	Eggs	Colobus monkey	Spain	–	Zoo	FM991955	–	Cutillas et al. [11]
*Trichuris discolour*	Adults	Japanese serow	Japan	–	In the wild	AB367795	–	Unpublished
*Trichuris discolor*	Adults	Bovine	Spain	–	Domestication	HE608846	–	Callejón et al. [60]
*Trichuris discolor*	Adults	Bovine	Iran	–	Domestication	FR870273	–	Callejón et al. [60]
*Trichuris discolor*	Adults	Bovine	Iran	–	Domestication	FR870272	–	Callejón et al. [60]
*Trichuris discolor*	Adults	Bovine	Spain	–	Domestication	HE608852	–	Callejón et al. [60]
*Trichuris discolor*	Adults	Wild yak	–	China	In the wild	–	NC_018596	Liu et al. [27]
*Trichuris muris*	Adults	Mouse	–	Japan	In the wild	–	AP017703	Unpublished
*Trichuris ovis*	Adults	Sheep	Ireland	–	Domestication	JF680987	–	Cavallero et al. [42]
*Trichuris ovis*	Adults	Sheep	Spain	–	Domestication	FR870274	–	Callejón et al. [60]
*Trichuris ovis*	Adults	Antelope	–	China	Zoo	–	NC_018597	Liu et al. [27]
*Trichuris suis*	Adults	Swine	China	–	Domestication	AM993003	–	Liu et al. [10]
*Trichuris suis*	Adults	Swine	China	–	Domestication	AM992999	–	Liu et al. [10]
*Trichuris suis*	Adults	Swine	China	–	Domestication	AM993007	–	Liu et al. [10]
*Trichuris suis*	Adults	Swine	China	–	Domestication	AM993012	–	Liu et al. [10]
*Trichuris suis*	Adults	Swine	China	–	Domestication	AM993004	–	Liu et al. [10]
*Trichuris suis*	Adults	Swine	–	Denmark	Domestication	–	KT449823	Hawash et al. [28]
*Trichuris suis*	Adults	Swine	–	China	Domestication	–	GU070737	Liu et al. [13]
*Trichuris trichiura*	Adults	Baboon	Turkey		Zoo	KC877992	–	Unpublished
*Trichuris trichiura*	Adults	Human	China	–		AM992984	–	Liu et al. [10]
*Trichuris trichiura*	Adults	Human	China	–		AM992981	–	Liu et al. [10]
*Trichuris trichiura*	Adults	Human	China	–		AM992997	–	Liu et al. [10]
*Trichuris trichiura*	Adults	Human	China	–		AM992994	–	Liu et al. [10]
*Trichuris trichiura*	Adults	Human	China	–		AM992990	–	Liu et al. [10]
*Trichuris trichiura*	Adults	Human	China	–		AM992991	–	Liu et al. [10]
*Trichuris trichiura*	Adults	Human	Cameroon	–		GQ301555	–	Ravasi et al. [23]
*Trichuris trichiura*	Adults	Human	–	China		–	GU385218	Liu et al. [13]
*Trichuris trichiura*	Adults	Human	–	Japan		–	AP017704	Unpublished
*Trichuris trichiura*	Adults	Human	–	Uganda		–	KT449826	Hawash et al. [28]
*Trichuris* sp.	Adults	Leaf monkey	China	–	Zoo	KT186233	–	Liu et al. [27]
*Trichuris* sp.	Adults	Leaf monkey	China	–	Zoo	KT186234	–	Liu et al. [27]
*Trichuris* sp.	Adults	Leaf monkey	China	–	Zoo	KT186231	–	Liu et al. [27]
*Trichuris* sp.	Adults	Leaf monkey	China	–	Zoo	KT186232	–	Liu et al. [27]
*Trichuris* sp.	Adults	Japanese macaque	Italy	–	Zoo	KP336469	–	Cavallero et al. [42]
*Trichuris* sp.	Adults	Japanese macaque	Italy	–	Zoo	KP336472	–	Cavallero et al. [42]
*Trichuris* sp.	Adults	Japanese macaque	Italy	–	Zoo	KP336467	–	Cavallero et al. [42]
*Trichuris* sp.	Adults	Japanese macaque	Italy	–	Zoo	KP336463	–	Cavallero et al. [42]
*Trichuris* sp.	Adults	Japanese macaque	Italy	–	Zoo	KP336471	–	Cavallero et al. [42]
*Trichuris* sp.	Adults	Japanese macaque	Italy	–	Zoo	KP336464	–	Cavallero et al. [42]
*Trichuris* sp.	Adults	Japanese macaque	Italy	–	Zoo	KP336465	–	Cavallero et al. [42]
*Trichuris* sp.	Adults	Japanese macaque	Italy	–	Zoo	KP336477	–	Cavallero et al. [42]
*Trichuris* sp.	Adults	Japanese macaque	Italy	–	Zoo	KP336470	–	Cavallero et al. [42]
*Trichuris* sp.	Adults	Chacma baboon	South Africa	–	In the wild	GQ301551	–	Ravasi et al. [23]
*Trichuris* sp.	Adults	Chacma baboon	South Africa	–	In the wild	GQ301552	–	Ravasi et al. [23]
*Trichuris* sp.	Adults	Chacma baboon	South Africa	–	In the wild	GQ301553	–	Ravasi et al. [23]
*Trichuris* sp.	Adults	Vervet monkey	Italy	–	Zoo	KP336481	–	Cavallero et al. [42]
*Trichuris* sp.	Adults	Vervet monkey	Italy	–	Zoo	KP336483	–	Cavallero et al. [42]
*Trichuris* sp.	Adults	Vervet monkey	Italy	–	Zoo	KP336484	–	Cavallero et al. [42]
*Trichuris* sp.	Adults	Golden snub-nosed monkey	China	–	Zoo	KT344825	–	This study
*Trichuris* sp.	Adults	Anubis baboon	China	–	Zoo	KT344826, MH390359-MH390361	–	This study
*Trichuris* sp.	Adults	Vervet monkey	China	–	Zoo	KT344827, MH390370	–	This study
*churis* sp.	Adults	Northern pig-tailed macaque	China	–	Zoo	KT344828, MH390364, MH390365	–	This study
*Trichuris* sp.	Adult	Rhesus monkey	China	–	Zoo	KT344829, MH390367-MH390369	–	This study
*Trichuris* sp.	Adults	Northern white-cheeked gibbon	China	–	Zoo	KT344830, MH390366	–	This study
*Trichuris* sp.	Adults	Hamadryas baboon	China	–	Zoo	KT344831, MH390363	–	This study
*Trichuris* sp.	Eggs	Black snub-nosed monkey	China	–	Zoo	KT344832, MH390362	–	This study
*Trichuris* sp.	Adults	Olive baboon	–	USA	Laboratory	–	KT449825	Hawash et al. [28]
*Trichuris* sp.	Adults	Hamadryas baboon	–	Denmark	Zoo	–	KT449824	Hawash et al. [28]
*Trichuris* sp.	Adults	Golden snub-nosed monkey	–	China	Zoo	–	KT344817	This study
*Trichuris* sp.	Adults	Anubis baboon	–	China	Zoo	–	KT344818,	This study
							MH390703-MH390705	
*Trichuris* sp.	Adults	Vervet monkey	–	China	Zoo	–	KT344819, MH390714	This study
*Trichuris* sp.	Adults	Northern pig-tailed macaque	–	China	Zoo	–	KT344820, MH390708	This study
							MH390709	
*Trichuris* sp.	Adults	Rhesus monkey	–	China	Zoo	–	KT344821,	This study
							MH390711-MH390713	
*Trichuris* sp.	Adults	Northern white-cheeked gibbon	–	China	Zoo	–	KT344822, MH390710	This study
*Trichuris* sp.	Adults	Hamadryas baboon	–	China	Zoo	–	KT344823, MH390707	This study
*Trichuris* sp.	Eggs	Black snub-nosed monkey	–	China	Zoo	–	KT344824, MH390706	This study
Outgroups								
*Trichinella spiralis*	Larvae	American black bear	USA	–	In the wild	KC006432	–	Unpublished
*Trichinella spiralis*	Larvae	Rat	–	USA	Laboratory	–	AF293969	Lavrov & Brown [40]
*Trichinella britovi*	Larvae	Mouse	–	Italy	Laboratory	–	KM357413	Mohandas et al. [41]

### Sequence and phylogenetic analysis

The nucleotide sequences of ITS and *cox*1 among species of *Trichuris* in the present study were edited with BioEdit (Ibis Biosciences, Carlsbad, USA) and subjected to separate alignment with reference sequences from closely related species (Table 2), including the congeneric species *T. trichiura*, *T. suis*, *T. muris*, *T. discolor*, *T. ovis* and *T. colobae* as well as two outgroup species *Trichinella spiralis* and *Trichinella britovi*, using the Clustal X 1.83 [33]. Codon-guided protein alignment manually adjusted the nucleotide alignment of *cox*1 during the procedure. Ambiguous sites/regions within these alignments were further filtered using GBlocks (http://molevol.cmima.csic.es/castresana/Gblocks_server.html) with default parameters. After Gblocks refining, both sequence alignment datasets were utilised for phylogenetic analyses using three different methods, namely neighbour-joining (NJ) (MEGA v.6.1 [34]), maximum parsimony (MP) (PAUP* 4.10b [35]) and Bayesian inference (BI) (MrBayes 3.2 [36]). NJ analysis was carried out in MEGA using either Kimura two-parameter (ITS) or Tamura-Nei (*cox*1) as the best-to-fit substitution model. For the MP analysis, heuristic searches were executed using tree-bisection-reconnection (TBR) branch-swapping algorithm, and 1000 random-addition sequence replicates with ten trees held at each step, and finally, the optimal topology with bootstrapping frequencies (BF) was obtained using Kishino-Hasegawa [37]. In the BI analysis, the general time reversible (GTR) including gamma-distributed rate variation (+G) and a proportion of invariable sites (+I) (= GTR + G + I; ITS) and gamma-distributed rate variation (= GTR + G; *cox*1) was determined as the best nucleotide substitution models using the Bayesian Information Criteria (BIC) test in jModeltest v. 2.1.6 [38], and the trees were constructed with the Markov chain Monte Carlo (MCMC) method (chains = 4) over 20,000,000 (ITS) or 500,000 (*cox*1) generations with every 20,000th (ITS) or 500th (*cox*1) tree being saved; when the average standard deviation of the split frequencies fell below 0.01, 25% of the first saved trees were discarded as “burn-in” and the consensus (50% majority rule) trees were inferred from the remaining trees and further visualised graphically with TreeviewX [39], with nodal supports expressed as posterior probabilities (PP). *T. spiralis* [40] alone or in combination with *T. britovi* [41] was used as outgroup reference and included in the phylogenetic analyses. Moreover, given almost identical nucleotide sequences of both ITS and *cox*1 genes observed in either worms or eggs from the same host species in this study, one representative specimen identified here was selected and coupled with *T. trichiura*, *T. suis*, *T. muris*, *T. discolor* and *T. ovis* as well as two putative species of *Trichuris* in baboon for further detection of synonymous and non-synonymous mutations in the mitochondrial *cox*1 gene based on their corresponding amino acid alignment, followed by determination of genetic distances between them using a distance matrix based on the maximum composite likelihood model in MEGA [37].

## Results

### Sequence characterisation

The final matrix of the ITS region obtained from each specimen of *Trichuris* collected from *R. roxellana*, *R. bieti*, *C. aethiops*, *M. mulatta*, *N. leucogenys*, *M. leonina*, *P. anubis* and *P. hamadryas*, respectively, was determined to be approximately 1200 bp in length after manual assembly. The sequences of the 5' and 3' ends of ITS of these *Trichuris* spp. were detected by comparison with those of *T. trichiura* from human (GenBank: AM992981) and *T. suis* from swine (GenBank: AM992999). The full lengths of the ITS region sequenced from these twenty *Trichuris* isolates according to the host as mentioned above order were as follows 1205 bp, 1220 bp, 1246 bp, 1248 bp, 1202 bp, 1241 bp, 1264 bp and 1202 bp, respectively, with a total 37.2–37.9% A + T content. The nucleotide sequences of ITS in either worms or eggs from the same host species were determined to be almost identical (99.9–100%). Sequence analysis revealed that *Trichuris* from *N. leucogenys* and *P. hamadryas* shared the highest identity (100%), followed by 99.8% identity between *Trichuris* from *R. roxellana* and *R. bieti* and 95.5–97.2% identity between *Trichuris* from *C. aethiops*, *M. leonina* and *P. anubis*. *Trichuris* from *M. mulatta* appeared to have the lowest sequence identity (91.8–94.6%) compared with the other isolates. Based on the identities, there were a total of 1203 conserved sites and 105 variable sites (including 99 parsimony-informative and six singleton sites) observed in the pairwise alignment of ITS. It was noteworthy that when the congeneric human whipworm *T. trichiura* was added into this alignment, *Trichuris* from *C. aethiops*, *N. leucogenys*, *M. leonina*, *P. anubis* and *P. hamadryas* consistently exhibited a higher identity with *T. trichiura* than that from *R. roxellana*, *R. bieti* and *M. mulatta* (data not shown). For the *cox*1 sequences, an identical sequence length (767 bp) was found across these 20 *Trichuris* isolates, with an average 62.9% A + T content, a typical mitochondrial nucleotide feature in nematodes (AT skewing). The base sequences of *cox*1 in either worms or eggs from the same host species were determined to be identical. Interestingly, nucleotide BLAST once again showed a high sequence identity (99.6%) occurring between *Trichuris* from *N. leucogenys* and *P. hamadryas*, the same as that of *Trichuris* from *R. roxellana* and *R. bieti*, although a higher identity (99.9–100%) was seen among *Trichuris* from the *C. aethiops*, *M. leonina* and *M. mulatta* (Additional file 1: Figure S1 and Additional file 2: Figure S2). Regarding identity comparisons, a total of 609 conserved sites and 158 variable sites (including 157 parsimony-informative and one singleton sites) were detected in the 767 bp pairwise alignment. Likewise, when the human-originated *T. trichiura* was introduced to the nucleotide alignment of *cox*1, species of *Trichuris* from *C. aethiops*, *N. leucogenys*, *M. leonina*, *P. anubis* and *P. hamadryas* and *M. mulatta* had higher identities (98.0–99.7%) with *T. trichiura* than that of *Trichuris* from *R. roxellana* (80.6%) and *R. bieti* (80.7%). Such an identity trend was also observed in their corresponding protein comparisons (not shown).

In this context, we specifically (i) located these conserved sites in *cox*1 by including other congeneric whipworms *T. suis*, *T. muris*, *T. discolor* and *T. ovis* as well as a *Trichuris* species in baboon *Trichuris* sp. and (ii) also focused on the variable sites, in order to determine if the base conservation was *Trichuris* lineage-specific and if there were non-synonymous substitutions apparent *via* comparison of their protein sequences in representative specimens. As shown in Additional file 1: Figures S1 and Additional file 2: Figure S2, we found that among 609 conserved base sites 455 were unique for the *Trichuris* lineage because no changes were observed across all congeneric species included in the alignment. Among 312 variable base sites, however, 204 were found unique for primate *Trichuris* spp. (in orange), including 66 being human *T. trichiura*-specific (in yellow) and 47 being non-human primate *Trichuris*-specific (in blue). Interestingly, among these 204 variable sites 98 were further found in two snub-nosed monkeys-specific (in red) and 24 were confirmed be non-synonymous substitutions based on protein alignments, which leaded to a total of 16 amino-acid changes, including V (Val) →I (Ilu), M (Met) → L (Leu), M (Met) → I (Ilu), F (Phe) → L (Leu), E (Glu) → N (Asn), Y (Tyr) → F (Phe), L (Leu) → I(Ilu), V (Val) → M (Met), M (Met) → T (Thr), S (Ser) → C (Cys), F (Phe) → L (Leu), S (Ser) →T (Thr), G (Gly) → S (Ser), S/T (Ser/Thr) →N (Asn) and I (Ilu) →V (Val) (see Additional file 2: Figure S2).

### Evolutionary distance analysis

The estimates of the evolutionary distance among these eight representative specimens of *Trichuris* and with other closely related whipworms were calculated and are shown in Table 3. Both datasets consistently placed species of *Trichuris* in *N. leucogenys* close to that in *P. hamadryas* and species of *Trichuris* in *R. roxellana* close to that in *R. bieti* with the minimum intraspecific evolutionary distances (0.0000 for ITS and 0.0030 for *cox*1 between the former; 0.0022 for ITS and 0.0030 for *cox*1 between the latter), in accordance with conclusions of our identity analysis. Among four other *Trichuris* isolates that were in *C. aethiops*, *M. leonina*, *P. anubis* and *M. mulatta*, respectively; however, our analysis indicated that their evolutionary distances varied by different genetic makers used. For example, *Trichuris* spp. in *M. leonina* and *M. mulatta* showed a maximum intraspecific evolutionary distance (0.0329) in ITS-based analysis while the value changed into 0.0000 in the *cox*1 data (see Table 3). Distances estimated between these eight representative specimens of *Trichuris* and other related congeners indicated the highest similarity to human *T. trichiura* and baboon *Trichuris* sp. with 0.0033–0.0317 (ITS) and 0.1352–0.2205 (*cox*1); more profound divergence characterised comparisons to *T. suis* with 0.3851–0.4043 (ITS) and 0.2338–0.2407 (*cox*1), *T. discolor* with 0.6098–0.6286 (ITS) and 0.2553–0.2705 (*cox*1) and *T. ovis* with 0.6175–0.6282 (ITS) and 0.2389–0.2755 (*cox*1) (Table 3).

**Table 3:** Estimates of evolutionary distance between *Trichuris* spp. recovered from different host species using nuclear ITS (below diagonal) and mitochondrial *cox*1 (above diagonal)

	Tsp_Nl	Tsp_Ph	Tsp_Pa	Tsp_Ca	Tsp_Ml	Tsp_Mm	Tsp_Rr	Tsp_Rb	Tsp	Ttr	Tsu	Tmu	Tdi	Tov	
Tsp_Nl		**0.0030**	**0.0144**	**0.0175**	**0.0165**	**0.0165**	**0.1882**	**0.1895**	0.1590	0.1423	0.2370	0.2275	0.2636	0.2771	Tsp_Nl
Tsp_Ph	**0.0000**		**0.0154**	**0.0186**	**0.0176**	**0.0176**	**0.1898**	**0.1911**	0.1575	0.1409	0.2370	0.2275	0.2636	0.2771	Tsp_Ph
Tsp_Pa	**0.0056**	**0.0056**		**0.0051**	**0.0040**	**0.0040**	**0.1911**	**0.1924**	0.1545	0.1352	0.2338	0.2243	0.2536	0.2740	Tsp_Pa
Tsp_Ca	**0.0078**	**0.0078**	**0.0067**		**0.0010**	**0.0010**	**0.1986**	**0.2000**	0.1586	0.1365	0.2387	0.2258	0.2569	0.2755	Tsp_Ca
Tsp_Ml	**0.0180**	**0.0180**	**0.0191**	**0.0191**		**0.0000**	**0.1972**	**0.1985**	0.1572	0.1352	0.2372	0.2243	0.2553	0.2740	Tsp_Ml
Tsp_Mm	**0.0340**	**0.0340**	**0.0352**	**0.0329**	**0.0329**		**0.1972**	**0.1985**	0.1572	0.1352	0.2372	0.2243	0.2553	0.2740	Tsp_Mm
Tsp_Rr	**0.0191**	**0.0191**	**0.0120**	**0.0120**	**0.0179**	**0.0340**		**0.0030**	0.2205	0.2004	0.2439	0.2365	0.2702	0.2421	Tsp_Rr
Tsp_Rb	**0.0191**	**0.0191**	**0.0202**	**0.0214**	**0.0260**	**0.0283**	**0.0022**		0.2174	0.1988	0.2407	0.2364	0.2705	0.2389	Tsp_Rb
Tsp	**0.0067**	**0.0067**	**0.0033**	**0.0033**	**0.0180**	**0.0317**	**0.0089**	**0.0214**		0.0489	0.2515	0.2435	0.2782	0.2808	Tsp
Ttr	0.0044	0.0044	0.0033	0.0056	0.0157	0.0317	0.0044	0.0168	0.0044		0.2330	0.2265	0.2483	0.2584	Ttr
Tsu	0.4043	0.4043	0.4019	0.3966	0.3883	0.3851	0.4004	0.3948	0.3983	0.3963		0.0635	0.2418	0.2546	Tsu
Tco	0.4228	0.4228	0.4203	0.4240	0.4178	0.4086	0.4188	0.4191	0.4185	0.4182	0.2246		0.2422	0.2552	Tmu
Tdi	0.6228	0.6228	0.6286	0.6217	0.6126	0.6098	0.6176	0.6194	0.6217	0.6211	0.5624	0.5699		0.1737	Tdi
Tov	0.6282	0.6282	0.6342	0.6272	0.6179	0.6175	0.6230	0.6236	0.6272	0.6266	0.5555	0.4185	0.0622		Tov
	Tsp_Nl	Tsp_Ph	Tsp_Pa	Tsp_Ca	Tsp_Ml	Tsp_Mm	Tsp_Rr	Tsp_Rb	Tsp	Ttr	Tsu	Tco	Tdi	Tov	

*Note*: Evolutionary distances between *Trichuris* spp. from eight non-human primates included in this study are highlighted in boldface for ITS- and *cox*1-based estimates, respectively. Given almost identical nucleotide sequences of both ITS and *cox*1 genes in either worms or eggs from the same host species, eight representative specimens were used to calculate evolutionary distances using a maximum composite likelihood model

*Abbreviations*: Tsp_Nl, *Trichuris* sp. from *Nomascus leucogenys*; Tsp_Ph, *Trichuris* sp. from *Papio hamadryas*; Tsp_Pa, *Trichuris* sp. from *Papio anubis*; Tsp_Ca, *Trichuris* sp. from *Chlorocebus aethiops*; Tsp_Ml, *Trichuris* sp. from *Macaca leonina*; Tsp_Mm, *Trichuris* sp. from *M. mulatta*; Tsp_Rr, *Trichuris* sp. from *Rhinopithecus roxellana*; Tsp_Rb, *Trichuris* sp. from *Rhinopithecus bieti*; Tsp, *Trichuris* sp.; Ttr, *Trichuris trichiura*; Tsu, *Trichuris suum*; Tco, *Trichuris colobae*; Tmu, *Trichuris muris*; Tdi, *Trichuris discolor*; Tov, *Trichuris ovis*

### Phylogenetic characterisation

**Fig. 2:**
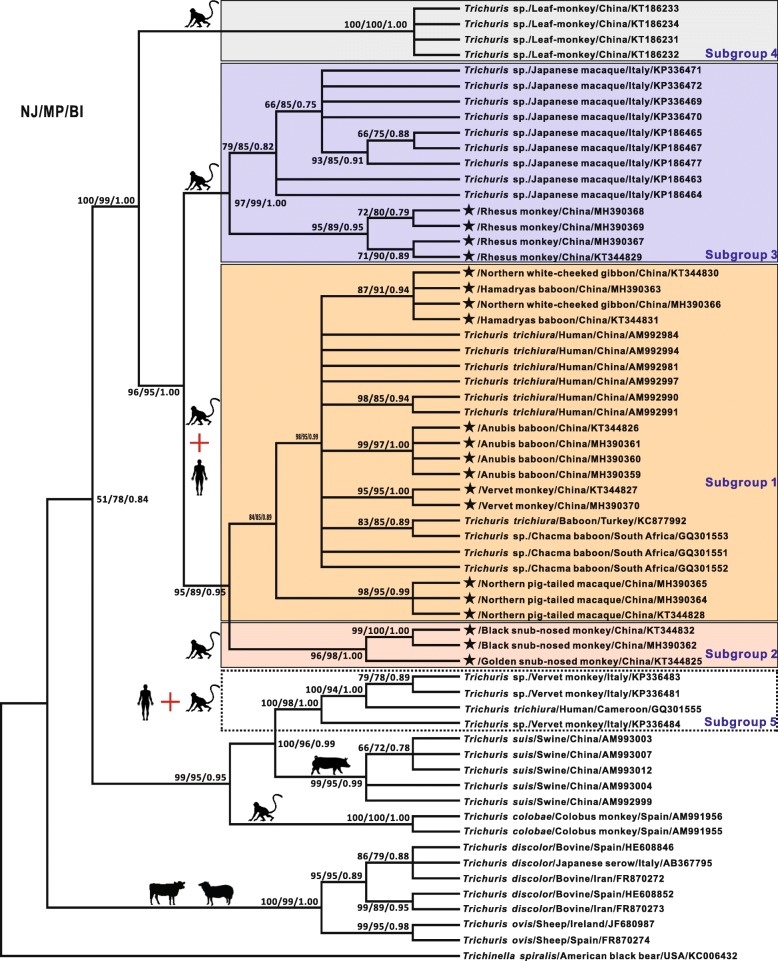
Inferred phylogenetic relationships of *Trichuris* spp. from primates and other mammals based on nuclear ITS1-5.8S-ITS2 sequences, with indications on host affiliation and assignment to subgroups. Phylogeny was inferred using neighbour joining (NJ), maximum parsimony (MP) and Bayesian inference (BI) methods. The reference species *Trichinella spiralis* was used as the outgroup. *Trichuris* spp. recovered from primates were genetically divided into five subgroups (Subgroups 1, 2, 3, 4 and 5) and indicated by differently coloured rectangles. The numbers along the branches show bootstrap values resulting from different analyses in the order NJ/MP/BI; values < 50% are not shown

**Fig. 3:**
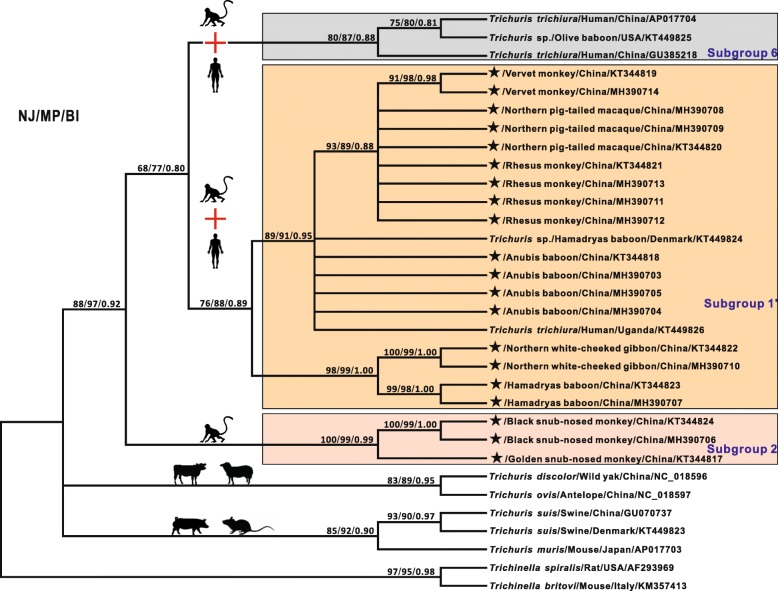
Inferred phylogenetic relationships of *Trichuris* spp. from primates and other mammals based on mitochondrial *cox*1 sequences, with indications on host affiliation and assignment to subgroups. Phylogeny was inferred using neighbour joining (NJ), maximum parsimony (MP) and Bayesian inference (BI) methods. The reference species *Trichinella spiralis* and *Trichinella britovi* were used as outgroups. Following the subgroup order and host affiliation in Fig. 2, *Trichuris* spp. recovered from primates were genetically divided into another two subgroups (Subgroups 1’ and 6) and indicated by differently coloured rectangles. The numbers along the branches show bootstrap values resulting from different analyses in the order NJ/MP/BI; values < 50% are not shown

The interrelationships of these 20 specimens of *Trichuris* and their phylogenetic relationships with other related species were inferred from the respective sequences of ITS and *cox*1 using NJ, MP and BI algorithms and their corresponding tree topologies are depicted in Figs. 2, 3. Although the two identical trees (NJ/MP/BI) topologically differed from each other because of genes and reference species included here, both analyses provided a consistent, robust phylogenetic resolution for these 20 isolates and their congeneric species in the genus *Trichuris.* Unequivocal subclades representing seven groups of *Trichuris* were demonstrated, showing varying patterns of broad to relatively narrow host range*.* Specifically, (i) *Trichuris* specimens in *N. leucogenys* and *P. hamadryas* clustered together and formed a sister cluster with other *Trichuris* in *C*. *aethiops*, *P*. *anubis* and *M*. *leonina* as well as other primates including humans, with robust support for both tree topologies (all bootstrap values ≥ 83 or 0.88); we named such clades as Subgroups 1 (Fig. 2) or 1’ (Fig. 3). (ii) *Trichuris* isolates in *R. roxellana* and *R. bieti* individually grouped as another clade with high statistical support (all values ≥ 96 or = 1.00) and were referred as Subgroup 2 in both ITS and *cox*1. (iii) *Trichuris* sp. in *M. mulatta* grouped with whipworms in the Japanese macaque *Macaca fuscata* Blyth, 1875 as a separate clade designated as Subgroup 3 in the ITS*-*based analysis but clustered with whipworms in *C*. *aethiops*, *M*. *leonina*, *P*. *anubis*, *P. hamadryas*, *N. leucogenys* and humans as an independent clade (Subgroup 1’) in the *cox*1 data, with a very similar host range to Subgroup 1 in the ITS. (iv) Four isolates of *Trichuris* in the François’ leaf monkey *Trachypithecus françoisi* (Pousargues, 1898) clustered together and formed a monophyletic group with all other primate whipworms included in this study and were referred as Subgroup 4 based on ITS data (Fig. 2). (v) Furthermore, one human-derived *Trichuris* grouped with the whipworms in vervet monkeys and together were closely related to *T. suis* and were referred to as Subgroup 5 in the ITS (Fig. 2), while two human-derived *Trichuris* grouped with the whipworm in a laboratory olive baboon and were named as Subgroup 6 in the *cox*1 (Fig. 3). (vi) For the inter-relationships of *Trichuris* spp. from these primates with *T. suis* from swine, *T. muri*s from mice, *T. discolor* from bovines and *T. ovis* from sheep, excluding Subgroup 5, their phylogenetic topologies were consistent with previously proposed molecular phylogenies of the whipworms based on the nuclear and mitochondrial DNA data [13, 26, 28, 42, 43], demonstrating the phylogenetic stability of these paraphyletic subgroups characterised in the present study.

## Discussion

Soil-transmitted *Trichuris* are among the most common parasitic nematodes found in mammals, including humans and non-human primates, and the etiological agent is usually considered as *T. trichiura*. Recent molecular epidemiological investigations, however, reveal that *T. trichiura* is not the only species of *Trichuris* to infect humans and non-human primates, suggesting the existence of a *Trichuris* species complex in primates. Given previously limited primate sampling and possible cross-infection and/or hybridisation among species of *Trichuris* [12, 29], the question about *Trichuris* as a large complex of distinct species rather than as a limited number of congeners in primates remains unclear. In the present study, the whipworms from an additional eight non-human primate hosts were included and combined with current existing data for *Trichuris* lineages in humans and other non-human primates.

Twenty whipworm isolates in this study were identified as *Trichuris* according to morphological characters [30, 31]. However, given current limited morphological diagnostic characters among *Trichuris* spp. [42] and a common phenomenon of morphological convergence in intestinal nematodes (e.g. *Ascaris* spp. [42, 44, 45] and *Anisakis* spp. [46]), the application of molecular markers for species identification was emphasised. For instance, the ITS of the nuclear ribosomal DNA is considered as a useful genetic marker for resolving nematode relationships at the species level [47]. Through pairwise comparisons of ITS among these twenty *Trichuris* isolates, we found that *Trichuris* spp. in *N. leucogenys* and *P. hamadryas* had an identical nucleotide sequence, suggesting that both hosts may harbour the same *Trichuris* species. A similar conjecture was also made for another pair of *Trichuris* spp. in *R. roxellana* and *R. bieti* due to the extremely high nucleotide identity (99.8%). To confirm these findings, ITS-based phylogenetic analyses (NJ/MP/BI) were performed and the results once again showed that species of *Trichuris* from the two pairs of hosts clustered together separately, with high bootstrap values (see Fig. 2), supporting the contention that *N. leucogenys* and *P. hamadryas* or *R. roxellana* and *R. bieti*, respectively, have their own *Trichuris* spp. Encouragingly, the same conclusions were further strengthened by analysis of another genetic marker, i.e. the mitochondrial *cox*1. It should be noted that *cox*1 analysis was added because numerous studies of nucleotide substitution in nematode mitochondrial genes (e.g. *cox*1 and *nad*4) have proven that they have utility in identifying and discovering novel or cryptic species among very closely related taxa due to their faster mutation rates relative to nuclear genes, maternal inheritance and lack of recombination [48–50]. Similar to the nuclear ITS, a comparable nucleotide identity (99.6%) was observed in *cox*1 of *Trichuris* spp. in either *N. leucogenys* and *P. hamadryas* or *R. roxellana* and *R. bieti*; more importantly, high bootstrap support was evident, based on phylogenetic analyses (NJ/MP/BI) of *cox*1 that demonstrated the same species of *Trichuris* in either *N. leucogenys* and *P. hamadryas* or *R. roxellana* and *R. bieti* (Additional file 2: Figure S2, Fig. 3). Nevertheless, possible cross-infection could be a confounder for this outcome because of the close or sympatric housing conditions in the zoos. This possibility was ruled out because sampling records, particularly for these two snub-nosed monkeys showed that *R. roxellana* was housed in the Chengdu Zoo (Sichuan, China) whereas *R. bieti* was kept in Kunming Zoo (Yunnan, China). Additionally, the captive history evidenced that these host populations were bred in the zoos and no translocations and/or introductions were reported before, suggesting that these *Trichuris* spp. were present with the original captive populations of these non-human primates and their infection cycles may have been maintained over time.

Based on the integrated molecular evidence, we propose that *Trichuris* spp. from either *N. leucogenys* and *P. hamadryas* or *R. roxellana* and *R. bieti* may represent the same species but belong to different genetic groups in the genus *Trichuris*, i.e. *Trichuris* sp. of *N. leucogenys* and *P. hamadryas* is within Subgroups 1 and 1’ and *Trichuris* sp. of *R. roxellana* and *R. bieti* is within Subgroup 2 (see Figs. 2, 3). Conversely, previous morphological studies showed that there were two different types of whipworms in *R. roxellana* and *R. bieti*. Specifically, *Trichuris rhinopiptheroxella* (Zhu et al., 2000) occurred in *R. roxellana* (also known Sichuan snub-nosed monkey) [51], and the congeneric species *Trichuris rhinopithecus* (Hou & Peng, 1989) occurred in *R. bieti* (also known Yunnan snub-nosed monkey) [52]. Both species of *Trichuris* were considered valid in terms of different lengths of vasa efferentia, ejaculatory duct, cloaca and spicules and locations of the ovary [51]. It seems remarkable that *T. rhinopiptheroxella* and *T. rhinopithecus* are heterogenic based on these morphological differences. However, current increased epigenetics-based evidence about phenotypic plasticity of parasites appears to be challenging the traditional morphological taxonomy [53]. Phenotypic plasticity is the concept that one genome can produce different phenotypes, usually in response to altered environmental conditions [54]. In fact, like vertebrates, invertebrate parasites also possess the ability to modify some aspects of their morphology and physiology adapting to different conditions including the external environment and inside environment of the host [55–57]. In such cases, phenotypic plasticity, based on morphometric criteria, has been purportedly expressed in many parasites. What is even more noteworthy is that phenotypic plasticity is particularly apparent among parasitic helminths because their range of hosts, origins and developmental stages can facilitate very different morphological and physiological characters useful for adaption and survival [58]. For example, within worms, Mati and colleagues [59] found that adult males of the human parasite *Schistosoma mansoni* maintained in AKR/J and Swiss mice, respectively, had significant morphometric changes in the total body length and reproductive characteristics (including the extension of testicular mass and number of testes) and that these morphometric alterations were also observed when compared to *S. mansoni* obtained from hamsters (*Mesocricetus auratus*), the host in which the parasite had been adapted and maintained for decades.

Nevertheless, all of these different phenotypes are products of the same genome including nuclear and mitochondrial DNAs. Thus, if taking the potential phenotypic plasticity of *Trichuris* spp. into account and combining with the aforementioned genetic evidence, our proposal that the same *Trichuris* species occurs in either *N. leucogenys* and *P. hamadryas* or *R. roxellana* and *R. bieti* is reasonable. Of course, additional information regarding ultrastructure and genomics of these *Trichuris* species and other related whipworms as well as broader taxonomic comparisons is still required to provide an increasingly precise morphological and molecular basis for species identification among whipworms. Additionally, there were 16 species-specific non-synonymous base substitutions detected in *cox*1 genes of *Trichuris* species in both *R. roxellana* and *R. bieti* (Additional file 2: Figure S2) that were further confirmed to be fixed after homologous comparisons with other congeneric species.

Phylogenetic analysis divided all primate *Trichuris* spp., including these 20 whipworms in this study, into seven distinct subgroups (Figs. 2, 3). Within ITS-based Subgroup 1, most whipworms were obtained from non-human primates (white-cheeked gibbons, baboons and monkeys) and clustered closely with either human-originated or baboon-derived *T. trichiura*. For instance, the whipworms in vervet monkey and anubis baboons shared a close relationship with human *T. trichiura* (Chinese isolates) than that of other non-human primates. Likewise, three specimens of *Trichuris* in chacma baboons from South Africa were also found to be more close to one baboon-derived *T. trichiura* (Turkey isolates) than others. Combined, these *Trichuris* spp. in chacma baboons from South Africa along with the whipworms in white-cheeked gibbon, hamadryas baboon, anubis baboon, vervet monkey and pig-tailed macaque from China were all grouped as part of the phylogenetically close *T. trichiura* clade. Such a conclusion would expand the host range of the ‘Clade DG’ as proposed by Ravasi et al. [23] and suggests that not only *T. trichiura* but multiple *Trichuris* species infect these host species. Referring to ITS-based Subgroup 1, since similar primate host cluster (excluding rhesus monkeys) was also found in *cox*1-based phylogeny we designated the *Trichuris* lineage as Subgroup 1’. In contrast to Subgroup 1, this subgroup placed *Trichuris* sp. in anubis baboons from China and *Trichuris* sp. in hamadryas baboon from Denmark more closely with human *T. trichiura* (Ugandan isolates) than *Trichuris* spp. in white-cheeked gibbon, hamadryas baboon, rhesus monkey, pig-tailed macaque and vervet monkey from China. This result is in agreement with a recently published study using partial (372 bp) *cox*1 sequences by Hawash et al. [28] and further supports that whipworms in primates comprise a *Trichuris* species complex. Under such hypothesis, compared with Subgroup 6, it seems that Subgroup 1’ reflects another fact that the human *Trichuris* from Uganda was genetically very distinct from that from China because both geographical isolates were found in two separate clades (see Fig. 3), suggesting at least two *Trichuris* spp. infecting humans. Besides, it is also noteworthy that among Subgroup 1’ *Trichuris* sp. in rhesus monkey clustered with that of pig-tailed macaque and vervet monkey while the rhesus monkey isolates grouped with whipworms in the Japanese macaque in ITS-based Subgroup 3. This discordance in the nuclear and mt phylogenies is surprising and may be owing to a higher rate of nucleotide change in mt DNA of nematodes than that seen in nuclear-encoded sequences, as reported in other *Trichuris* spp. [60], *Caenorhabditis* spp. [61] and *Baylisascaris* spp. [62].

Also, Subgroup 2 comprising two whipworms in *R. roxellana* and *R. bieti*, Subgroup 4 containing four specimens of *Trichuris* sp. in François’ leaf monkeys and Subgroup 5 having three vervet monkey-derived *Trichuris* and one human-derived *Trichuris*, three clades appear to be three independent lineages. These clades are genetically distant from other *Trichuris* species in white-cheeked gibbon, baboons and pig-tailed macaque, suggesting different species of *Trichuris* exist among non-human primates, as recently reported by Hawash et al. [28] and Liu et al. [27]. Previous studies have shown that there may be several *Trichuris* species parasitising primates, with some species only infecting non-human primates and others infecting both non-human primates and humans. Variation in host range reflects the interaction of trends in generalisation and specialisation (oscillation), the opportunity for host colonisation and capacity to establish infection as reflected in outcomes for ecological fitting in sloppy fitness space [63, 64] and more broadly the recently proposed Stockholm Paradigm (e.g. [65, 66]). Faunal mixing and the potential for exchange are facilitated in zoo-park situations that may bring phylogenetically disparate assemblages of hosts and their parasites into proximity. For parasites with a direct life-cycle such as *Trichuris*, this establishes the potential for considerable opportunities for host colonisation that may not be apparent in natural settings. A breakdown of mechanisms for ecological isolation as demonstrated in zoo-park environments emphasise the potential for extensive colonisation processes that may result and the possibility of new disease through the exchange of parasites over time. Moreover, ITS- or *cox*1-based phylogenies consistently showed that *T. trichiura*-included clades include Subgroups 1, 1’, 5 and 6. This finding should enhance public alertness to whipworms in these hosts and raise concerns about animal and human health.

As a part of epidemiological surveys of captive non-human primates and species diversity among associated *Trichuris* whipworms in Chinese zoos, a number of questions are apparent: (i) what are the potential routes of parasite introduction and establishment (natural or cross infections); (ii) what is the most appropriate pathway for surveillance of parasite infections, particularly when different cryptic species of helminths may be involved; (iii) what are the most appropriate strategies to prevent parasite transport in the space-limited and to an extent artificial zoos; (iv) what are the limits and facilitators for zoonotic risk. Given that the recognised host range (defined by opportunity) of a parasite is almost always a reduced subset of the actual host range (defined by capacity to use host-based resources) that is possible, the current study provides some quasi-experimental insights about the role of breakdown in isolation and ecological fitting in expansions of host range. These are characteristics most often associated with patterns of emerging infectious diseases in the context of ecological disruption, suggesting direct lessons about the potential versus realised current host range for many species of *Trichuris* and the risk of zoonotic infections. Historically within the assemblage of *Trichuris* which circulate in humans and non-human primates, bouts of host colonisation and oscillations in host range, appear as prominent contributors to faunal assembly and diversity [66, 67]. Therefore, there is an urgent need to clarify the *Trichuris* species which infect primates in order to discover transmission routes and establish suitable control measures.

## Conclusions

In this study, based on analyses of the nuclear ITS and mitochondrial *cox*1 datasets, we suggest at least seven genetically distinct subgroups of *Trichuris* present among primates including humans, supporting a previous proposal that a complex of *Trichuris* species other than *T. trichiura* infects these hosts. Furthermore, combined evidence of genetic distance analysis and phylogenies revealed that an identical whipworm species might exist either between the white-cheeked gibbon and hamadryas baboon or Sichuan and Yunnan snub-nosed monkeys. However, such a proposal requires further examination based on broad sampling and extensive morphological and genetic comparisons. Taken together, the results presented in this study once again emphasise that the taxonomy and genetics of *Trichuris* spp. in primates are complex and these cumulative molecular and phylogenetic data should contribute to a better understanding of the taxonomy, genetics and evolutionary biology of the whipworms.

## Additional files

Additional file 1: **Figure S1.** Alignment of the nucleotide sequence of mitochondrial *cox*1 genes of eight representative isolates of *Trichuris* identified in this study and congeneric species. Nucleotide sequences of *cox*1 genes were retrieved from the GenBank database and aligned using the Clustal X 1.83 software. Species abbreviations and accession numbers (in parentheses) are indicated as follows: Tsp_Ph (DK), *Trichuris* sp. from *Papio hamadryas* (KT449824); Tsp_Ph (CN), *Trichuris* sp. from *Papio hamadryas* (KT344823); Tsp_Nl *Trichuris* sp. from *Nomascus leucogenys* (KT344822); Tsp_Pa, *Trichuris* sp. from *Papio anubis* (KT344818); Tsp_Ml, *Trichuris* sp. from *Macaca leonie* (KT344820); Tsp_Mm, *Trichuris* sp. from *M. mulatta* (KT344821); Tsp_Ca *Trichuris* sp. from *Chlorocebus aethiops* (KT344819); Tsp_Rr *Trichuris* sp. from *Rhinopithecus roxellana* (KT344817) and Tsp_Rb *Trichuris* sp. from *Rhinopithecus bieti* (KT344824); Ttr, *Trichuris trichiura* (GU385218); Tsu, *Trichuris suum* (GU070737); Tmu, *Trichuris muris* (AP017703); Tdi, *Trichuris discolor* (NC_018596); Tov, *Trichuris ovis* (NC_018597). The two abbreviations DK and CN denote different geographical origins, Denmark and China, respectively. Pairwise comparisons between *Trichuris* spp. identified in this study were highlighted in grey. (PDF 381 kb)

Additional file 2: **Figure S2.** A simultaneous alignment of nucleotide and amino acid sequences of mitochondrial *cox*1 genes of eight representative isolates of *Trichuris* identified in this study and their congeneric species. Building on alignments from Figure S1, the corresponding amino acid sequences inferred according to the Invertebrate Mitochondrial Code were added and aligned. Regions of identity in either nucleotide (.) or amino-acid (:) are indicated. The variable base loci unique for primate *Trichuris* spp. are highlighted in orange; among those is human *T. trichiura*-specific that are highlighted in yellow and non-human primate *Trichuris*-specific highlighted in blue. Further, 98 variable base loci unique for two snub-nosed monkeys are identified (in red) in order to test the non-synonymous substitutions, and a total of 16 amino-acid changes: V (Val)/I (Ilu), M (Met)/L (Leu), M (Met)/I (Ilu), F (Phe)/L (Leu), E (Glu)/N (Asn), Y (Tyr)/F (Phe), L (Leu)/I (Ilu), V (Val)/M (Met), M (Met)/T (Thr), S (Ser)/C (Cys), F (Phe)/L (Leu), S (Ser)/T (Thr), G (Gly)/S (Ser), S/T (Ser/Thr)/N (Asn) and I (Ilu)/V (Val) are observed and targeted with a black star. Percentages of nucleotide identities between these eight representative isolates of *Trichuris* are shown at the end of each sequence, with a number tag: (1) *Trichuris* sp._Ph, (2) *Trichuris* sp._Pa, (3) *Trichuris* sp._Nl, (4) *Trichuris* sp._Ml, (5) *Trichuris* sp._Mm, (6) *Trichuris* sp._Ca, (7) *Trichuris* sp._Rr, (8) *Trichuris* sp._Rb. (PDF 420 kb)

## Abbreviations

BI: Bayesian inference
BIC: Bayesian Information Criteria
*cox*1: Cytochrome *c* oxidase subunit 1
ITS: Internal transcribed spacers
MCMC: Markov chain Monte Carlo
MP: Maximum parsimony
mtDNA: Mitochondrial DNA
NJ: Neighbor-joining
PCR: Polymerase chain reaction
PP: Posterior probabilities
STHs: Soil-transmitted helminths
TBR: Tree-bisection-reconnection
