# Supplementary figures and images for: Genetic characterisation and phylogenetic status of whipworms (Trichuris spp.) from captive non-human primates in China, determined by nuclear and mitochondrial sequencing
Source: Parasit Vectors. 2018 Sep 20;11:516. doi: 10.1186/s13071-018-3100-5 (PMC6149069; doi:10.1186/s13071-018-3100-5)

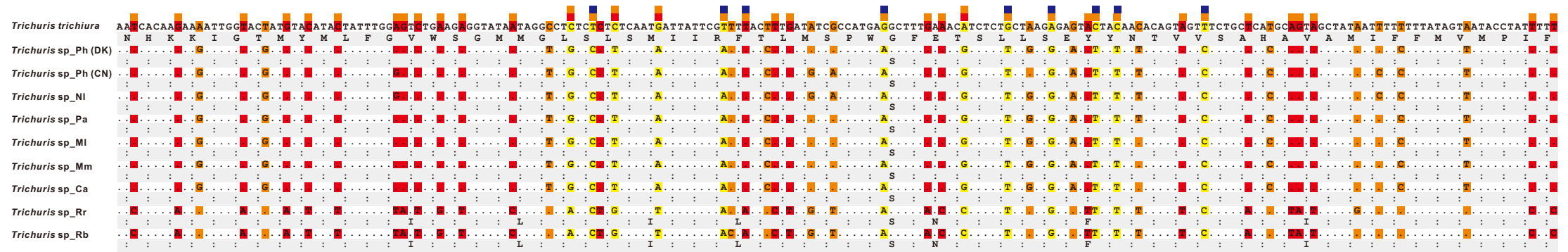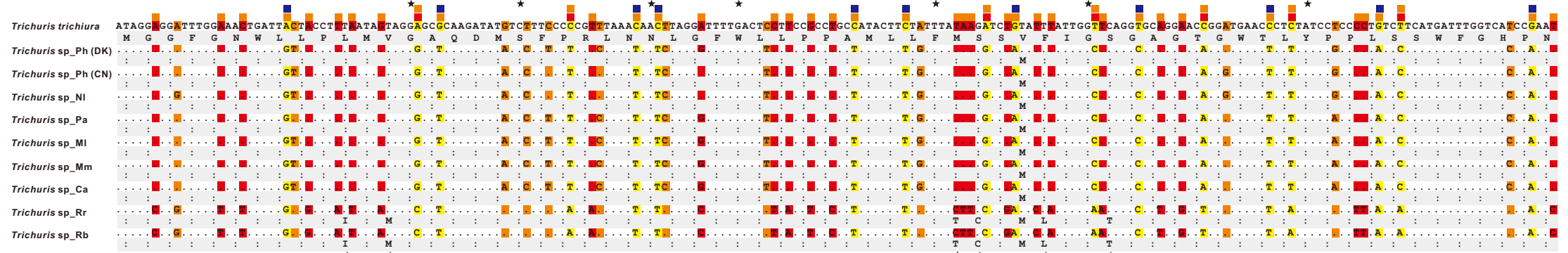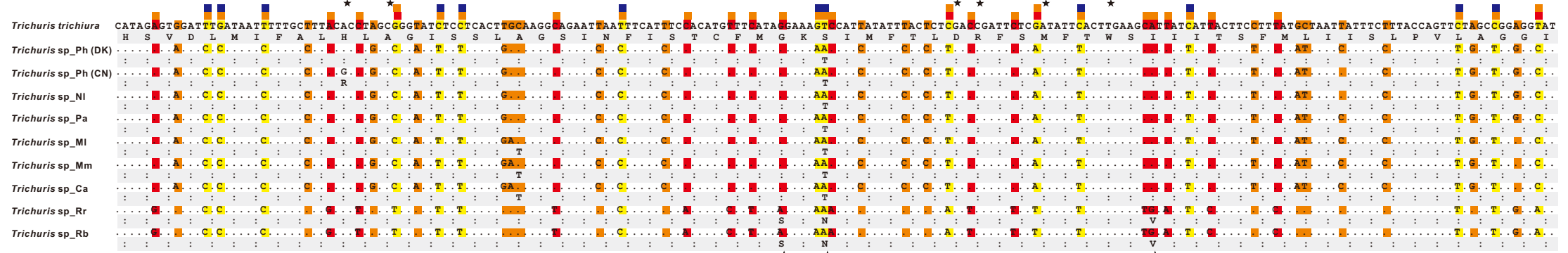[illegible]

Supplement: Supplementary file 2 — Figure S2. A simultaneous alignment of nucleotide and amino acid sequences of mitochondrial cox1 genes of eight representative isolates of Trichuris identified in this study and their congeneric species. Building on alignments from Figure S1, the corresponding amino acid sequences inferred according to the Invertebrate Mitochondrial Code were added and aligned. Regions of identity in either nucleotide (.) or amino-acid (:) are indicated. The variable base loci unique for primate Trichuris spp. are highlighted in orange; among those is human T. trichiura-specific that are highlighted in yellow and non-human primate Trichuris-specific highlighted in blue. Further, 98 variable base loci unique for two snub-nosed monkeys are identified (in red) in order to test the non-synonymous substitutions, and a total of 16 amino-acid changes: V (Val)/I (Ilu), M (Met)/L (Leu), M (Met)/I (Ilu), F (Phe)/L (Leu), E (Glu)/N (Asn), Y (Tyr)/F (Phe), L (Leu)/I (Ilu), V (Val)/M (Met), M (Met)/T (Thr), S (Ser)/C (Cys), F (Phe)/L (Leu), S (Ser)/T (Thr), G (Gly)/S (Ser), S/T (Ser/Thr)/N (Asn) and I (Ilu)/V (Val) are observed and targeted with a black star. Percentages of nucleotide identities between these eight representative isolates of Trichuris are shown at the end of each sequence, with a number tag: (1) Trichuris sp._Ph, (2) Trichuris sp._Pa, (3) Trichuris sp._Nl, (4) Trichuris sp._Ml, (5) Trichuris sp._Mm, (6) Trichuris sp._Ca, (7) Trichuris sp._Rr, (8) Trichuris sp._Rb. (PDF 420 kb) [file 13071_2018_3100_MOESM2_ESM.pdf]
