# Supplementary material for: Genetic characterisation and phylogenetic status of whipworms (Trichuris spp.) from captive non-human primates in China, determined by nuclear and mitochondrial sequencing
Source: Parasit Vectors. 2018 Sep 20;11:516. doi: 10.1186/s13071-018-3100-5 (PMC6149069; doi:10.1186/s13071-018-3100-5)
Supplement: Supplementary file 1 — Figure S1. Alignment of the nucleotide sequence of mitochondrial cox1 genes of eight representative isolates of Trichuris identified in this study and congeneric species. Nucleotide sequences of cox1 genes were retrieved from the GenBank database and aligned using the Clustal X 1.83 software. Species abbreviations and accession numbers (in parentheses) are indicated as follows: Tsp_Ph (DK), Trichuris sp. from Papio hamadryas (KT449824); Tsp_Ph (CN), Trichuris sp. from Papio hamadryas (KT344823); Tsp_Nl Trichuris sp. from Nomascus leucogenys (KT344822); Tsp_Pa, Trichuris sp. from Papio anubis (KT344818); Tsp_Ml, Trichuris sp. from Macaca leonie (KT344820); Tsp_Mm, Trichuris sp. from M. mulatta (KT344821); Tsp_Ca Trichuris sp. from Chlorocebus aethiops (KT344819); Tsp_Rr Trichuris sp. from Rhinopithecus roxellana (KT344817) and Tsp_Rb Trichuris sp. from Rhinopithecus bieti (KT344824); Ttr, Trichuris trichiura (GU385218); Tsu, Trichuris suum (GU070737); Tmu, Trichuris muris (AP017703); Tdi, Trichuris discolor (NC_018596); Tov, Trichuris ovis (NC_018597). The two abbreviations DK and CN denote different geographical origins, Denmark and China, respectively. Pairwise comparisons between Trichuris spp. identified in this study were highlighted in grey. (PDF 381 kb) [file 13071_2018_3100_MOESM1_ESM.pdf]

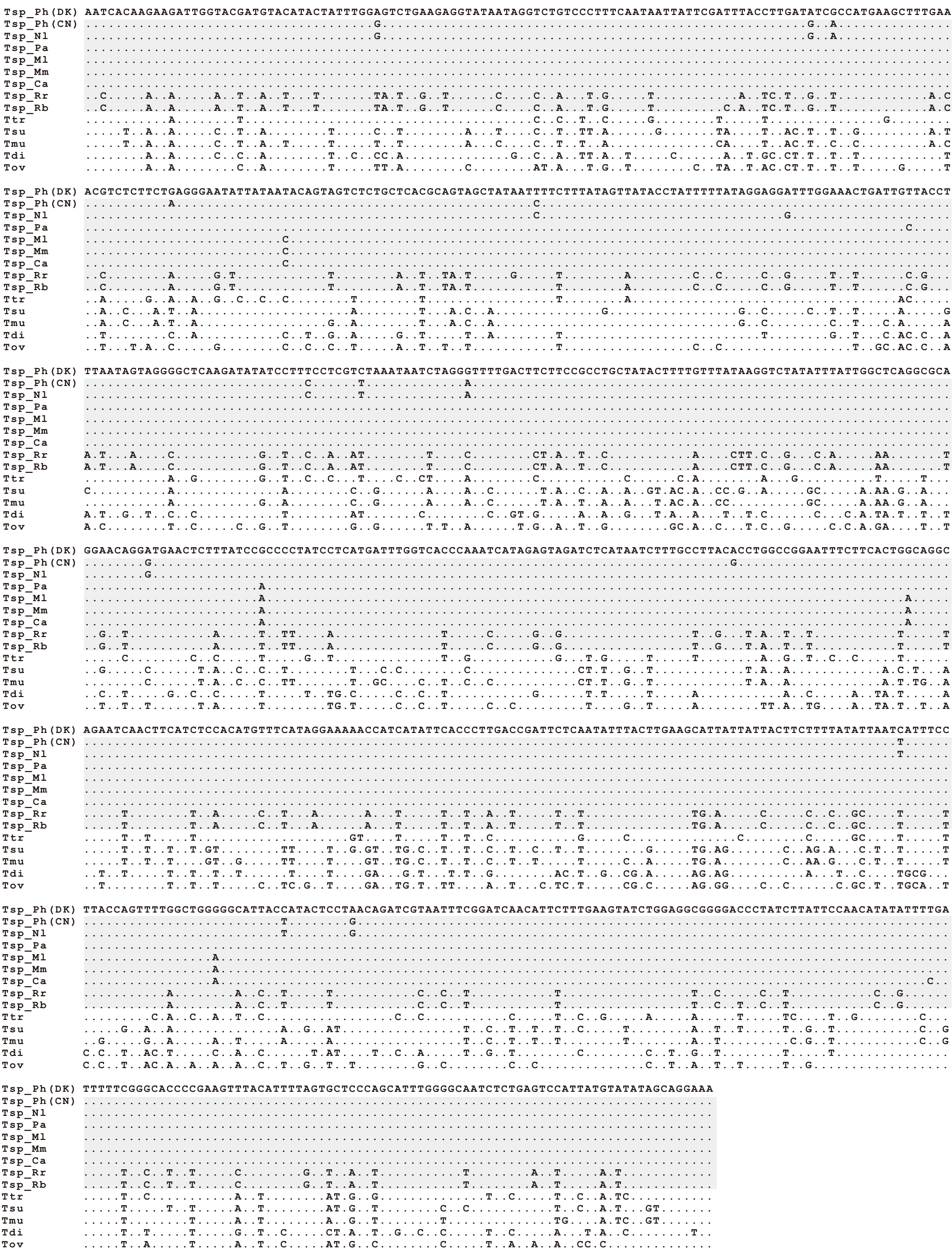
Tsp_Ph(DK)
Tsp_Ph(CN)
Tsp_Nl
Tsp_Pa
Tsp_Ml
Tsp_Mm
Tsp_Ca
Tsp_Rr
Tsp_Rb
Ttr
Tsu
Tmu
Tdi
Tov
